# Supplementary material for: A qualitative study of COVID-19 vaccine intentions and mistrust in Black Americans: Recommendations for vaccine dissemination and uptake
Source: PLoS One. 2022 May 3;17(5):e0268020. doi: 10.1371/journal.pone.0268020 (PMC9064113; doi:10.1371/journal.pone.0268020)
Supplement: Attachment — Submitted filename: Response_to_reviews_R2.docx [file pone.0268020.s002.docx]

**Reviewer #2:**

The revisions have strengthened the paper. Some suggestions that might strengthen it further:

1. I would encourage the authors to consider whether some of the in-text quotations are too truncated, making them poor representations of the ideas they are claimed to represent. Examples are on page 14 "looking for profit" and page 15 "fear and anxiety".

**Thank you for the additional suggestions. We have removed these short, truncated in-text quotations, and changed them into full quotations as appropriate (please see pages 13-16, marked version).*

2. In-text quotations disappear after page 17. There are good quotations in the table that speak to the content, but it might be nice to include something in the latter half of the findings as it makes the findings section unbalanced.

**We have included selected exemplar quotes in text on pages 17-20 (marked version).*

3. I would strongly recommend reconsidering the claim on page 22: “our results also indicated challenges that are attributed to racial identity and other vulnerabilities and marginalized identities such as socioeconomic status and sexual/gender identities.” There was no gender or sexual identity analysis visible in the results section, therefore, this reads as speaking beyond the results. The point is a good one but would be better identified as something to be pursued in future research. The findings do reference financial barriers and that could be discussed in the discussion, with support of relevant literature.

**We have revised the sentence (on gender or sexual identity) to discuss a future research direction (page 24, paragraph 3, marked version). In addition, we added a discussion point on financial barriers (page 21, paragraph 1, marked version).*
